# Application of Machine Learning Algorithm in Predicting Axillary Lymph Node Metastasis from Breast Cancer on Preoperative Chest CT

**DOI:** 10.3390/diagnostics13182953

**Published:** 2023-09-14

**Authors:** Soyoung Park, Jong Hee Kim, Yoon Ki Cha, Myung Jin Chung, Jung Han Woo, Subin Park

**Affiliations:** 1 Department of Health Sciences and Technology, SAIHST, Sungkyunkwan University, Seoul 06351, Republic of Korea; thyoung94@gmail.com (S.P.); subinn.park@gmail.com (S.P.); 2 Department of Radiology, Samsung Medical Center, Sungkyunkwan University School of Medicine, Seoul 06351, Republic of Korea; jonghk1101@naver.com (J.H.K.); jhwoo9984@gmail.com (J.H.W.)

**Keywords:** breast cancer, machine learning, axilla, lymph nodes, lymphatic metastasis, computed tomography

## Abstract

Axillary lymph node (ALN) status is one of the most critical prognostic factors in patients with breast cancer. However, ALN evaluation with contrast-enhanced CT (CECT) has been challenging. Machine learning (ML) is known to show excellent performance in image recognition tasks. The purpose of our study was to evaluate the performance of the ML algorithm for predicting ALN metastasis by combining preoperative CECT features of both ALN and primary tumor. This was a retrospective single-institutional study of a total of 266 patients with breast cancer who underwent preoperative chest CECT. Random forest (RF), extreme gradient boosting (XGBoost), and neural network (NN) algorithms were used. Statistical analysis and recursive feature elimination (RFE) were adopted as feature selection for ML. The best ML-based ALN prediction model for breast cancer was NN with RFE, which achieved an AUROC of 0.76 ± 0.11 and an accuracy of 0.74 ± 0.12. By comparing NN with RFE model performance with and without ALN features from CECT, NN with RFE model with ALN features showed better performance at all performance evaluations, which indicated the effect of ALN features. Through our study, we were able to demonstrate that the ML algorithm could effectively predict the final diagnosis of ALN metastases from CECT images of the primary tumor and ALN. This suggests that ML has the potential to differentiate between benign and malignant ALNs.

## 1. Introduction

Being the most common cancer in women worldwide, breast cancer poses a great challenge to public health on a global scale [1]. Axillary lymph node (ALN) status is one of the most critical prognostic factors for patients with breast cancer [2]. In fact, identification of the presence of ALN metastasis plays a pivotal role in pathologic staging, prognosis, and guidance of treatment in patients with breast cancer [3].

Although widespread adaptation of MRI and PET/CT in breast cancer staging has improved sensitivity and specificity in the assessment of lymph node metastasis compared with CT, the long scan time may increase patients’ discomfort [4]. The greater number of contraindications associated with MRI and the higher cost of a PET/CT scan are also obstacles to wide usage compared with other diagnostic modalities [4]. More significantly, chest contrast-enhanced CT (CECT) not only displays enhanced breast cancer tissue and ALN metastasis, but also excludes possible intrapulmonary and thoracic bone metastases [5]. As a result, chest CECT has been widely used for preoperative evaluation of breast cancer [5,6,7]. However, according to previous studies using CT and MRI to differentiate benign from malignant lymph nodes, there have been limitations in assessing accurate size or signal intensity threshold [8]. Despite its limitations in differentiating benign and malignant lymph nodes, CT is still a widely used modality. There is a great need for non-invasive and accurate methods for predicting ALN metastasis in breast cancer.

In the era of artificial intelligence, current developments in radiology focus on the improvement of decision support systems to maximize the potential role of non-invasive imaging modalities [9]. In the previous study by Zhou et al., they developed a deep learning algorithm to predict ALN metastasis in patients with clinically negative ALN using the US images of primary breast cancers [10]. The best-performing CNN achieved 85% sensitivity and 73% specificity compared to the 73% sensitivity and 63% specificity of the radiologists [10]. Recently, Chen et al. developed a CNN model to predict sentinel lymph node status based on dynamic contrast-enhanced MRI [11]. Their CNN model showed the best performance in tumor groups smaller than 0.2cm with an AUC of 0.081 at the internal validation set and 0.823 at the external test set 1 [11].

Furthermore, there have been a few studies predicting ALN metastasis based on preoperative CT images [12]. In the previous study by Yang et al., they developed a deep learning model with only features of ALN itself for the prediction of ALN metastasis [12]. However, according to the most widely used monograms and scoring system for predicting ALN metastasis in clinical practice [13,14,15,16,17,18,19], primary tumor characteristics, such as tumor size, are also considered, as well as features of ALN [20]. Therefore, the aim of our study was to investigate the potential of the machine learning (ML) model for predicting ALN metastasis with chest CECT, using not only the features of ALN itself but also the primary tumor.

## 2. Materials and Methods

### 2.1. Data Collection

**Figure 1:**
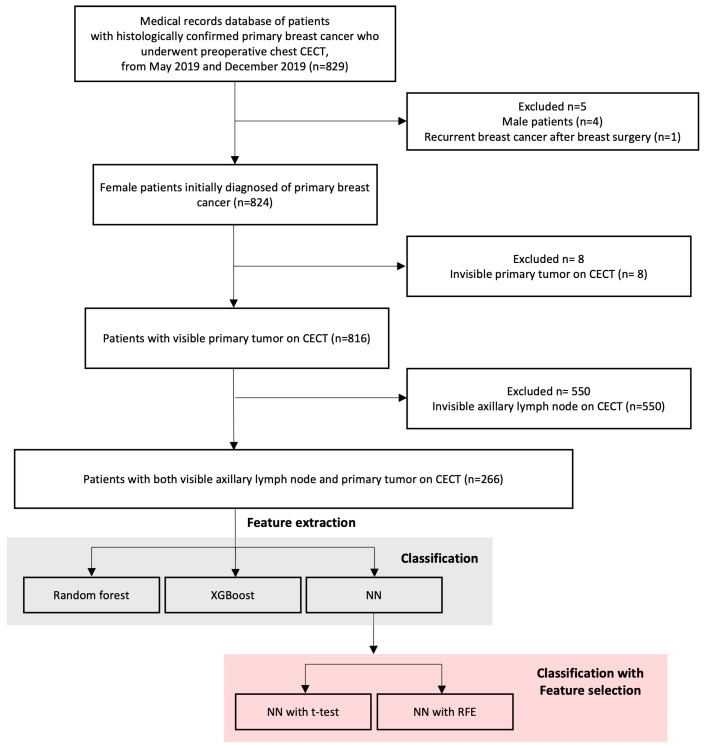
Flowchart of selecting study subjects for the development and evaluation of machine learning models for axillary lymph node metastasis prediction. NN: Neural network. RFE: recursive feature elimination.

The institutional review board (IRB) of Samsung Medical Center approved this retrospective study. The requirement for patient consent to use clinical data was waived by the IRB due to a retrospective study design (IRB file number: 2021-08-031). Between May 2019 and December 2019, 829 patients with breast cancer who underwent preoperative chest CECT with both pre- and post-contrast-enhancement protocols were included in this study. Inclusion criteria were (1) pathologically confirmed breast cancer patients who underwent preoperative chest CECT and (2) those who had no history of previous neoadjuvant chemotherapy. Exclusion criteria were as follows: (1) No visible primary tumor on chest CECT; (2) no visible suspicious ALN on chest CECT; (3) male patients; and (4) recurrent breast cancer after breast surgery. Finally, a total of 266 patients were selected for this study. The flow chart of the selection of study subjects is shown in Figure 1.

### 2.2. Feature Extraction

We collected age of patients as a demographic feature. Imaging features of primary tumor and ALN were collected by two thoracic radiologists with 16 and 30 years of experience. 

The location of the primary tumor (T_site; left or right) and the maximal diameter of the primary tumor (T_size) were evaluated. Pattern and degree of enhancement of the primary tumor were also evaluated. Since there is no generally used standard for breast imaging or classification with chest CECT, we evaluated enhancement pattern of the primary tumor based on 5th BI-RADS lexicon for MRI [21]. Although enhancement characteristics of masses are divided into six types on BI-RADS lexicon, we reduced them into three categories. Since MRI provides greater soft tissue visualization than CT, detailed enhancement patterns, such as rim enhancement and enhancing internal septations, might only appear as heterogeneous enhancements on CT [22]. Therefore, enhancement patterns were divided into the following three categories: T_homogeneous, T_heterogeneous, and T_non-mass. 

T_homogeneous stands for uniform and confluent enhancement throughout the mass, which is same as BIRADS MRI lexicon [21]. T_heterogeneous is an integrated category of several BIRADS MRI lexicons, including heterogeneous enhancement, rim enhancement, and enhancing internal septations [21]. T_non-mass is defined as an enhancement that has neither a tri-dimensional mass nor typical mass characteristics [21]. 

Within T_homogeneous category, enhancement degree was also evaluated. It was categorized as follows: if the degree of contrast enhancement of primary tumor was similar to that of the most adjacent vessel, it was considered as a marked enhancement; if the enhancement degree of the tumor was higher than that of the ipsilateral latissimus dorsi or serratus anterior muscle, but lower than that of the vessel, it was considered a moderate degree; if the enhancement degree was similar to that of the ipsilateral latissimus dorsi or serratus anterior muscle, it was considered a mild enhancement; and if it was lower than that of the ipsilateral latissimus dorsi or serratus anterior muscle, it was considered a minimal enhancement [23]. To avoid subjective bias of reading features, two radiologists examined and marked a region of interest (ROI) to sign both primary tumor and ALN with the highest metastatic potential [23,24].

**Figure 2:**
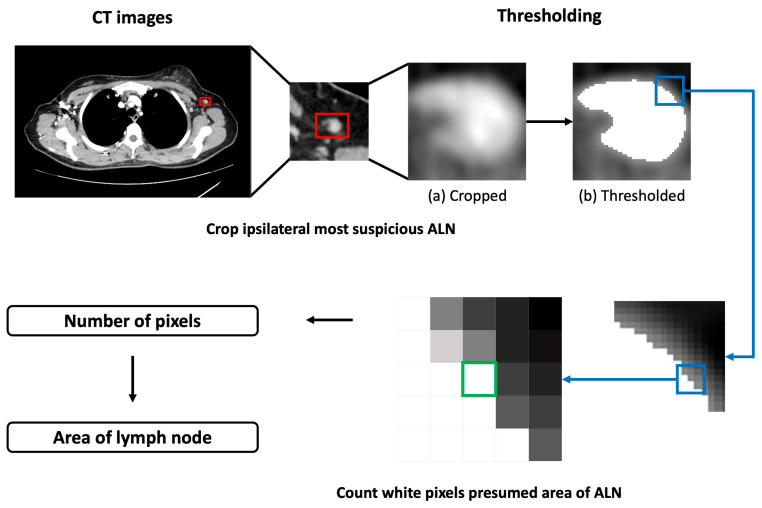
Flowchart of the measurement of the area of the lymph node suspected of metastasis. The red rectangle is the region of interest (ROI) of the most suspicious axillary lymph node (ALN). The green box is one of the white pixels.

We extracted features by measuring Hounsfield unit (HU) value for the primary tumor, aorta, and ALN. T_average (average HU value of primary tumor) was measured as region of interest covering more than half of the tumor diameter. T_SD was the standard deviation (SD) of HU values of the primary tumor. A_average was the average HU value of attenuation of aorta. A_SD was SD of HU values of aorta. N_average was the average HU value in the most suspected ALN metastasis in CECT. N_area was the approximate area of the most suspected ALN. Workflow of N_area measurement is described in Figure 2.

We cropped the most suspicious ALN from isometric resampled CECT image. We then determined the threshold of the cropped image. Values above the thresh were set to be maxValue. Otherwise, values were unchanged, as shown in the following equation:

$$thresholded\left(x,\;y\right)=\left\{\begin{array}{cc} maxValue & if\;cropped\left(x,\;y\right)>thresh\\ cropped\left(x,\;y\right) & otherwise \end{array}\right.$$

where $cropped\left(x,\;y\right)$ was the HU value for each pixel of cropped ALN image, thresh was the threshold value, and $thresholded\left(x,\;y\right)$ was the thresholded image.

Next, the number of pixels in maxValue was counted. The value of maxValue used was white, which was (255, 255, 255) in RGB. Since we performed isometric resampling, the number of pixels could refer to the ALN area.

Lastly, since the degree of contrast enhancement might be affected by intrinsic factors, such as cardiac output and BMI, to eliminate the influence of intrinsic factors, we applied aorta-based corrected attenuation value [25]. Therefore, three variables, Δ(Aorta − Tumor), Δ(Aorta − Node), and Δ(Tumor − Node) were generated by calculating each difference between three variables, A_average, T_average, and N_average.

Consequently, we collected a total of 15 variables (Age, T_site, T_size, T_homogeneous, T_heterogeneous, T_non-mass, T_average, T_SD, N_average, N_area, A_average, A_SD, Δ(Aorta − Tumor), Δ(Aorta − Node), and Δ(Tumor − Node)) for each patient. There was no missing value for any variable.

To analyze collected variables with ML, feature encoding methods were applied. For categorical variables in which there was no ordinal relationship, a one-hot encoding technique was used. For categorical features with ordinal relationships, they were encoded into zero and non-zero variables.

### 2.3. Feature Selection Methods

Feature selection is one of the most important processes that can improve the performance and efficiency of computation time. This is accomplished by selecting a subset of features that could derive an optimal performance of the model in the entire feature set while eliminating irrelevant, redundant, and noisy features that do not contribute to the model’s performance [26,27]. If the number of data used for training is small, it might be difficult for the model to learn important relationships sufficiently [28]. Therefore, by using feature selection, we tried to facilitate the learning process with a limited amount of data while removing less important features [29]. 

We applied statistical methods and recursive feature elimination (RFE) to exclude the least important features and identify the meaningful feature subset used in the neural network (NN) model. Regarding statistical methods, a *t*-test was used for numerical variables and a chi-square test was used for categorical variables. These tests were performed using SPSS software for Windows ver. 27.0 (SPSS Inc., Chicago, IL, USA). For both statistical analyses, features with *p*-values less than 0.05 were regarded as statistically different between metastasis and non-metastasis. 

We gathered only statistically significant variables and created one subset. The RFE could eliminate the least meaningful features iteratively until the area under the curve (AUC) reached the maximum value [26,30,31]. In the iterative process in RFE, one can gain the best feature subset that provides the highest AUC. RFE was performed using Python software (version 3.7.11).

### 2.4. Machine Learning Algorithms

Three ML algorithms were used to compare performances of models for classifying whether there was any breast cancer metastasis to ALN: random forest (RF) [32], extreme gradient boosting (XGBoost) [33], and NN. RF is an ensemble learning algorithm for classification, regression, and other tasks with a combination of decision trees that calculate the mode or mean/average of each tree. XGBoost is also an ensemble algorithm that builds multiple gradients boosted decision trees to maximize efficiency and performance [33]. NN algorithm mimics the operation of the human brain [34]. It is constructed with an input layer, an output layer, and singular or multiple hidden layers placed between two of them. 

We tuned hyperparameters of NN via grid search at each RFE step to achieve better performance [35,36]; so, a learning rate of 0.001 for Adam optimizer [37], batch size of 10, and epochs of 300 were used. In the same way, hyperparameters of RF and XGBoost were also tuned. These ML-driven predictions of breast cancer metastasis to ALN were developed using TensorFlow (version 2.3.0), Keras (version 2.4.0), Scikit-learn (version 0.24.2) libraries, and Python software (version 3.7.11).

**Figure 3:**
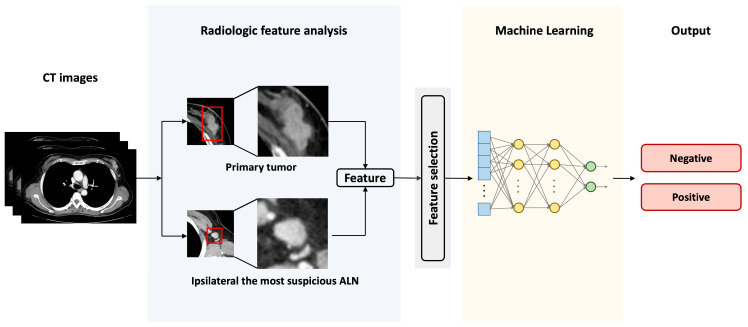
Schematic illustration of the machine learning based prediction framework for axillary lymph node (ALN) metastasis of breast cancer. The red rectangle is the region of interest (ROI) of the primary tumor and the most suspicious axillary lymph node (ALN).

For more advanced experiments, we applied two feature selection algorithms, statistical analysis and RFE, to NN. The final workflow for predicting ALN metastasis is shown in Figure 3.

### 2.5. Evaluation of Machine Learning Models

To develop ML algorithms, the total dataset was split into a training set and a test set. The training set was used to teach breast cancer metastasis to ALN prediction algorithms, and the test set was used to evaluate trained algorithms. At the training phase, we used stratified 10-fold cross-validation. This randomly divided the dataset into 10 partitions (folds) while keeping the ratio of each label (e.g., positive and negative) distribution for each fold. We then repeatedly validated and evaluated the model using 9 partitions as training data and the remaining 1 partition as validation data. These techniques assured a model’s generalized performance and prevented overfitting [38]. Because outputs of ML-driven algorithms were probabilistic estimates of breast cancer metastasis in ALN, the performance for each model was evaluated using area under the receiver operating characteristic curve (AUROC) and confusion matrix, comparing them with histopathological examination results of ALN.

## 3. Results

### 3.1. Data Collection and Feature Engineering

All 266 patients were females with a median age of 50 years (range, 23–81 years). Among the 266 patients with breast cancer and most suspicious ALN metastasis, 186 patients had ALN metastasis while 80 patients did not have ALN metastasis. Table 1 shows demographics and characteristics of the collected dataset. 

T_site did not show significant differences between metastatic and benign ALN groups. (p = 0.593). T_size and N_area were significantly associated with ALN metastasis (p < 0.05). Regarding the enhancement pattern of the primary tumor, 234 patients had T_homogeneous features (87.97%), 8 patients had T_heterogeneous features (3.01%), and 24 patients had T_non-mass features (9.02%). In the T_homogeneous group, 3 patients had a minimal enhancement, 33 patients had a mild enhancement, 115 patients had a moderate enhancement, and 83 patients had a marked enhancement. Average and SD of HU values were 85.96 ± 22.02 (T_average) and 11.71 ± 5.84 (T_SD) for the primary tumor and 174.39 ± 21.46 (A_average) and 8.65 ± 2.32 (A_SD) for the aorta of all patients. None of these four features was associated with ALN metastasis. On the other hand, among the two features extracted from ALN, N_area (884.01 ± 282.24, p < 0.05) was statistically significant. Three features that presented a difference in enhancement degree did not show meaningful characteristics in statistical analysis.

### 3.2. Performance Comparisons of ML Models for Detecting ALN Metastasis

Among three classification models which trained all features, NN recorded higher AUROC (0.71 ± 0.13) than RF (0.63 ± 0.11) and XGBoost (0.62 ± 0.08). NN with statistical analysis showed a lower AUROC (0.63 ± 0.12) performance than NN but similar to RF and XGBoost. The NN with RFE reached the highest AUROC of 0.76 ± 0.11 and accuracy of 0.74 ± 0.12 when compared to three classification models without feature selection and to another NN model with feature selection (NN with statistical analysis). Table 2 summarized results of five experiments for classifying ALN metastasis.

All 15 features were inserted into RF, XGBoost, and NN. For classification with feature selection, four features (T_size, T_homogeneous, T_non-mass, and N_area) selected via statistical feature selection were used for NN with stats, and eight features (T_size, T_average, N_average, N_area, A_average, Δ(Aorta − Tumor), Δ(Aorta − Node), and Δ(Tumor − Node)) selected via RFE were used for NN with RFE.

To validate the importance of ALN-related features, we also tried the NN with RFE model without ALN-related features. The results of the performance comparison of NN with ALN features and NN without ALN features are shown in Table 3. Since there were four ALN-related features (N_average, N_area, Δ(Aorta − Node), and Δ(Tumor − Node)), the number of input variables for RFE without lymph features was 11.

**Figure 4:**
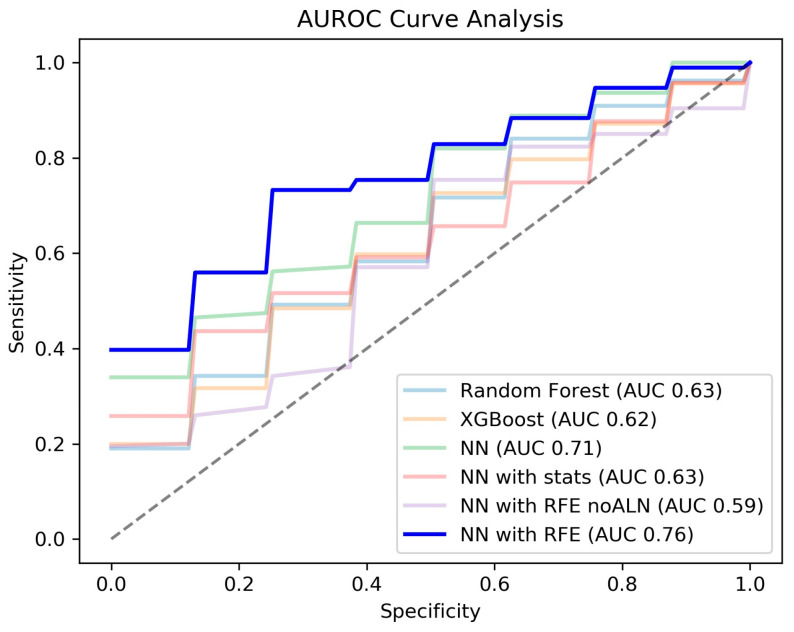
Receiver operating characteristic (AUROC) curve of the six tested algorithms.

During RFE without lymph features experiment, the best performance was shown when there were four input features (T_size, T_average, A_average, and Δ(Aorta − Tumor)). Compared with RFE with lymph features, all evaluation variables of RFE with lymph features showed higher performances. Especially, AUROC and Specificity increased the most from 0.59 ± 0.17 to 0.76 ± 0.11 and 0.71 ± 0.25 to 0.88 ± 0.12. Figure 4 shows the AUROC curves of all six tested ML algorithms.

## 4. Discussion

Despite its clinical importance, the prediction of lymph node metastasis is still challenging in the field of imaging. As shown in our study, radiologic features between benign and malignant ALN evaluated by two radiologists showed ambiguous results. There was no significant difference in the average enhancement degree between benign and malignant lymph nodes. Furthermore, the sizes of primary tumor and lymph nodes were even larger in benign ALN groups. This finding might be partly due to a selection bias caused by our radiologic assessing method, which evaluates the largest suspicious lymph nodes, even for benign ALN cases.

Our ambiguous result regarding the size and enhancement degree between benign and malignant ALN is well correlated with the previous studies on limitations of CT and MRI in the evaluation of metastatic lymph nodes [8]. Although previous studies which differentiated metastatic lymph nodes based on size using CT had controversial results, they generally showed low accuracy [8,39,40]. Furthermore, several studies have investigated the utility of dynamic contrast enhancement for differentiating normal from metastatic lymph nodes [8,41,42,43]. Although they observed a significantly longer time to peak, lower peak enhancement, and slower washout slope for malignant lymph nodes, they also indicated the difficulty in standardizing acquisition parameters to obtain reproducible data [8]. This might be due to the fact that conventional human vision evaluation is dependent on the knowledge and familiarity of the radiologists. 

Various ML approaches have been proposed for classifying or detecting breast cancer metastasis in ALN [44,45,46]. Song et al. [45] proposed a ML-based radiomics model for predicting ALN metastasis in invasive ductal breast cancer (IDC). The volume of interests (VOIs) were drawn in the primary tumor on the PET scan, and texture features were extracted in 100 consecutive IDC patients who underwent surgical resection of the primary tumor and/or ALN dissection. They used XGBoost to select features and evaluate the model. Zheng et al. [44] developed a deep learning radiomics (DLR) model that classified the ALN extent in early stage breast cancer. They used the US, sheer wave elastography (SWE) images, and clinicopathological data of 584 patients. Their combined DLR model offers a non-invasive imaging biomarker which can predict the status of ALN for patients with early stage breast cancer. Zhang et al. [46] developed a multiparametric MRI model combined with ensemble learning to predict ALN metastasis in invasive breast cancer. They fused axial T2WI, DWI, and DCE-MRI models and achieved an AUC of 0.913. 

In this study, we trained ML models to predict the ALN metastasis of breast cancer patients using clinical information and imaging features of both the primary tumor and ALN on preoperative chest CECT images. Additionally, in order to avoid overfitting, when we trained models directly from images using a small amount of data, we extracted handcrafted knowledge-based features and adapted them for training.

The best performance was achieved by NN with RFE, and it reached an AUROC of 0.72 ± 0.09 and an accuracy of 0.64 ± 0.15. Interestingly, our experiments showed that the RFE provides a better feature subset to improve the performance of NN than statistical analysis with our dataset. Statistical methods, such as t-test and chi-square, could be used to compare means and analyze if there is any probable difference between two groups. Since features are analyzed independently, feature dependencies could be ignored, leading to a poor feature subset [47]. On the other hand, since RFE uses sequential elimination, it interacts with classifiers with the advantage of capturing feature dependencies [48].

To understand the importance of ALN-related features for predicting ALN metastasis, we conducted a comparative experiment. As a result of comparing the performance of the NN with the RFE model along with or without ALN-related features from chest CECT, all performance measurements increased, showing that analyzing both primary tumor and ALN has a positive effect on predicting ALN metastasis. Particularly, specificity increased from 0.71 ± 0.25 to 0.88 ± 0.12. Due to the increase in specificity via the addition of CECT features of ALNs and primary tumors, unnecessary treatment processes should be avoidable, and procedure-related side effects may also be prevented [49,50].

Our work has notable strengths compared with other previous studies predicting breast cancer metastasis using ML models. To the best of our knowledge, most of the data types or methods used in previous studies could be divided into two types: (1) multiple image modalities, such as ultrasonography, X-ray, CT, or MRI, for extracting features in a single area (either primary tumor, lymph node, or other organs); and (2) multiple image modalities, such as ultrasonography, X-ray, CT, or MRI, for extracting features in multiple areas (one image modality for each area) [12,44,51,52]. However, unlike previous studies, we attempted to predict metastasis by considering both the primary tumor and ALN in chest CECT via the extraction of features from two areas using a single image modality [12]. Compared to multimodal image, single modal image research has fewer training parameters, so it benefits in computational cost and is less limited in building layers and channels [53].

This study has several limitations. Firstly, we used the experimental dataset from a single institution to train and evaluate the ML model. It is often difficult to gather a large-scale medical image dataset due to issues such as annotation cost or privacy [54,55]. However, since chest CECT images may differ depending on the scanner, performance may deteriorate if external validation is performed. Moreover, two radiologists went through the additional process of labeling the pattern and the degree of enhancement of the primary tumor while conducting this study, which means it required a lot of annotation cost to use unstructured readings for training. Furthermore, pathologic and imaging correlations were not performed for ALNs one by one. In cases of multiple ALNs on the ipsilateral side of the primary tumor, we only evaluated the most suspicious ALN. Therefore, collected imaging features of lymph node might show ambiguous results regarding lymph node size and enhancement. Finally, several cases with non-visible primary tumor or lymph nodes on CT were excluded. Although primary tumor and lymph nodes are not delineated on CT, such cases might also have pathologically confirmed lymph node metastasis. Characteristic findings of those cases were not evaluated in this study.

In further studies with expanded data collections, security and privacy issues should be considered for a more widespread application in the clinical setting [56]. Also, to increase the utilization of additionally collected data, it is necessary to go through the process of generating structured readings [57] by finding important keywords that are used repeatedly in the readings of various radiologists [57,58].

## 5. Conclusions

In conclusion, we adopted ML methods to classify ALN metastasis of breast cancer patients using clinical information and chest CECT image features. Although we used a limited amount of data, we were able to demonstrate that our ML models could classify ALN metastasis of breast cancer by using appropriate feature selection methods considering both primary tumor and ALN features. Imaging features of both ALNs and primary tumors from chest CECT play an important role in ML models for the ALN metastasis of breast cancer. With further validation in a larger population and model calibration, our ML technique might serve as an important decision support tool in clinical applications when predicting ALN metastasis in breast cancer.

## Figures and Tables

**Table 1:** Demographics and characteristics of the collected dataset.

			All Patients	ALN Metastasis (−)	ALN Metastasis (+)	*p*-Value
		Age (years)	50 (23–81)	49.5 (23–77)	50 (32–81)	-
**Primary tumor**						
	T_site (left/right)		133:133	38:42	95:91	-
	T_size (mm)		18.72 ± 10.03	21.27 ± 13.54	17.62 ± 7.85	<0.05
	**Enhancement pattern**					
		T_homogeneous ^a^ (E_1_:E_2_:E_3_:E_4_)	234 (87.97%) (3:33:115:83)	65 (81.25%) (1:13:30:21)	169 (90.86%) (2:20:85:62)	<0.05
		T_heterogeneous	8 (3.01%)	3 (3.75%)	5 (2.69%)	-
		T_non-mass	24 (9.02%)	12 (15.00%)	12 (6.45%)	<0.05
	**Enhancement degree (HU)**					
		T_average	85.96 ± 22.02	88.78 ± 24.64	84.75 ± 20.74	-
		T_SD	11.71 ± 5.84	11.26 ± 5.79	11.90 ± 5.87	-
**Lymph node**						
	N_average		81.44 ± 21.31	83.68 ± 21.46	80.48 ± 21.23	-
	N_area		884.01 ± 282.24	940.35 ± 243.31	859.78 ± 294.73	<0.05
**Aorta**						
	A_average		174.39 ± 21.46	173.17 ± 19.24	174.92 ± 22.38	-
	A_SD		8.65 ± 2.32	8.33 ± 2.10	8.78 ± 2.40	-
**Calibrated enhancement degree ^b^**						
	Δ(Aorta − Tumor)		88.43 ± 26.34	84.39 ± 27.11	90.17 ± 25.89	-
	Δ(Aorta − Node)		92.95 ± 26.13	89.49 ± 22.09	94.44 ± 27.60	-
	Δ(Tumor − Node)		4.52 ± 27.16	5.11 ± 28.87	4.27 ± 26.48	-

Values are presented as number of patients, median (range), number of patients per category separated by colons, or mean value ± standard deviation. ALN: axillary lymph node; HU: Hounsfield Unit. ^a^ Categorized by the degree of enhancement of primary tumor. (E_1_) Minimal enhancement. (E_2_) Mild enhancement. (E_3_) Moderate enhancement. (E_4_) Marked enhancement. ^b^ Enhancement degree corrected through the elimination of intrinsic factors, such as BMI and cardiac output.

**Table 2:** Performance comparison of machine learning models for predicting ALN metastasis.

	Classification			Classification with Feature Selection	
	**RF**	**XGBoost**	**NN**	**NN** **with Stats ^a^**	**NN** with RFE *
**AUROC**	0.63 ± 0.11 (0.47–0.84)	0.62 ± 0.08 (0.51–0.74)	0.71 ± 0.13 (0.49–0.84)	0.63 ± 0.12 (0.49–0.87)	**0.76 ± 0.11** **(0.61–0.97)**
**Accuracy**	0.70 ± 0.07 (0.56–0.81)	0.66 ± 0.06 (0.56–0.74)	0.72 ± 0.12 (0.44–0.85)	0.61 ± 0.13 (0.44–0.85)	**0.74 ± 0.12** **(0.56–0.92)**
**PPV**	0.72 ± 0.04	0.73 ± 0.04	0.90 ± 0.09	0.92 ± 0.07	**0.93 ± 0.06**
**NPV**	0.48 ± 0.35	0.45 ± 0.23	0.61 ± 0.20	0.45 ± 0.11	**0.59 ± 0.19**
**Sensitivity**	0.91 ± 0.08	0.82 ± 0.08	0.71 ± 0.24	0.49 ± 0.21	**0.69 ± 0.29**
**Specificity**	0.18 ± 0.13	0.28 ± 0.13	0.75 ± 0.24	0.88 ± 0.12	**0.88 ± 0.12**

Values are mean ± standard deviation of 10-fold cross-validation results and values in the bracket are minimum–maximum values of 10-fold cross-validation results. RF: random forest; NN: neural network; RFE: recursive feature elimination; AUROC: area under the receiver operating characteristic curve; PPV: positive predictive value; NPV: negative predictive value. ^a^ NN with statistical analysis. * RFE with lymph features (same model as used in Table 3).

**Table 3:** Classification performances with or without lymph node features for predicting ALN metastasis.

	RFE without Lymph Features	RFE with Lymph Features *
**AUROC**	0.59 ± 0.17 (0.34–0.96)	**0.76 ± 0.11** **(0.61–0.97)**
**Accuracy**	0.66 ± 0.18 (0.37–0.88)	**0.74 ± 0.12** **(0.56–0.92)**
**PPV**	0.88 ± 0.11	**0.93 ± 0.06**
**NPV**	0.53 ± 0.17	**0.59 ± 0.19**
**Sensitivity**	0.63 ± 0.32	**0.69 ± 0.29**
**Specificity**	0.71 ± 0.25	**0.88 ± 0.12**

Values are mean ± standard deviation of 10-fold cross-validation results and values in the bracket are minimum–maximum values of 10-fold cross-validation results. AUROC: area under the receiver operating characteristic curve; PPV: positive predictive value; NPV: negative predictive value. * NN with RFE (same model as used in Table 2).

## Data Availability

The datasets generated during this study are not publicly available due to privacy and ethical considerations; however, anonymized data can be provided by the corresponding author upon reasonable request and with the approval of the ethics committee. Researchers interested in accessing the data should contact the corresponding author for further information.
